# Modified ‘one amino acid-one codon’ engineering of high GC content TaqII-coding gene from thermophilic *Thermus aquaticus* results in radical expression increase

**DOI:** 10.1186/1475-2859-13-7

**Published:** 2014-01-11

**Authors:** Agnieszka Zylicz-Stachula, Olga Zolnierkiewicz, Katarzyna Sliwinska, Joanna Jezewska-Frackowiak, Piotr M Skowron

**Affiliations:** 1 Department of Molecular Biotechnology, Faculty of Chemistry, University of Gdansk, Wita Stwosza 63, 80-308 Gdansk, Poland

## Abstract

**Background:**

An industrial approach to protein production demands maximization of cloned gene expression, balanced with the recombinant host’s viability. Expression of toxic genes from thermophiles poses particular difficulties due to high GC content, mRNA secondary structures, rare codon usage and impairing the host’s coding plasmid replication.

TaqII belongs to a family of bifunctional enzymes, which are a fusion of the restriction endonuclease (REase) and methyltransferase (MTase) activities in a single polypeptide. The family contains thermostable REases with distinct specificities: TspGWI, TaqII, Tth111II/TthHB27I, TspDTI and TsoI and a few enzymes found in mesophiles. While not being isoschizomers, the enzymes exhibit amino acid (aa) sequence homologies, having molecular sizes of ~120 kDa share common modular architecture, resemble Type-I enzymes, cleave DNA 11/9 nt from the recognition sites, their activity is affected by S-adenosylmethionine (SAM).

**Results:**

We describe the *taqIIRM* gene design, cloning and expression of the prototype TaqII. The enzyme amount in natural hosts is extremely low. To improve expression of the *taqIIRM* gene in *Escherichia coli* (*E. coli*), we designed and cloned a fully synthetic, low GC content, low mRNA secondary structure *taqIIRM*, codon-optimized gene under a bacteriophage lambda (λ) *P*_*R*_ promoter. Codon usage based on a modified ‘one amino acid–one codon’ strategy, weighted towards low GC content codons, resulted in approximately 10-fold higher expression of the synthetic gene. 718 codons of total 1105 were changed, comprising 65% of the *taqIIRM* gene. The reason for we choose a less effective strategy rather than a resulting in high expression yields ‘codon randomization’ strategy, was intentional, sub-optimal TaqII *in vivo* production, in order to decrease the high ‘toxicity’ of the REase-MTase protein.

**Conclusions:**

Recombinant wt and synthetic *taqIIRM* gene were cloned and expressed in *E. coli*. The modified ‘one amino acid–one codon’ method tuned for thermophile-coded genes was applied to obtain overexpression of the ‘toxic’ *taqIIRM* gene. The method appears suited for industrial production of thermostable ‘toxic’ enzymes in *E. coli.* This novel variant of the method biased toward increasing a gene’s AT content may provide economic benefits for industrial applications.

## Background

Thermophilic bacteria, which thrive at temperatures greater than 50°C, require special adaptation strategies at the genome, transcriptome and proteome levels. The pattern of synonymous codon usage within thermophilic prokaryotes is different from that within mesophilic ones [1-6]. This difference is the result of natural selection linked to thermophily [1,6]. Differences in codon usage between species adversely affect recombinant gene expression levels, thus gene optimization is often needed to obtain adequate expression levels, which is especially important for industrial enzyme production processes. Natural REase-coding genes found in wild-type (wt) organisms are often not highly expressed, due to the ‘toxicity’ of their protein product to their hosts, if not fully protected by cognate MTases. The subtle balance between both enzymatic activities, comprising the restriction-modification (RM) system, can be affected by environmental conditions and lead to the cell’s death, caused by genome damage. Moreover, this problem is much more pronounced in a recombinant host, harbouring the cloned RM system, due to the different coding gene regulatory circuits. Recent development in artificial gene synthesis has enabled the construction of synthetic genes [7-10], and thus made possible the rational design of artificial genes and their functional clusters, described as a ‘synthetic biology’ approach. Synthetic biology can be used to overcome problems of low gene expression in heterologous hosts, which is a crucial economical aspect in industrial gene expression. Although the gene expression is highly correlated with codon usage, the problem is not as simply defined or solved. A general preference for the use of codons of the highest frequency in the genome or in the highly expressed gene subset of the host is not necessarily a guarantee of improved expression [10,11].

To aid the gene design process, computational tools have been developed [12]. Typically, two strategies have been used for codon optimization. The first one, known as ‘one amino acid–one codon’ assigns the most abundant codon of the recombinant host or a set of selected genes to a given amino acid (aa) in the target sequence [13]. The second, ‘codon randomization’, uses translation tables, based on the frequency distribution of the codons in a genome or a subset of highly expressed genes. Each codon has an assigned weight or probability. As a result, a random mixture of codons assigned for a given aa is used to assemble the synthetic gene. In this case, as codons are assigned randomly, a vast number of possible gene variants can be obtained [13]. This allows for further nt sequence fine-tuning, without altering the final aa sequence. Many of the accessible sequence design software tools are focused on the frequency of Individual Codon Occurrences (ICU) as one of the most crucial factors affecting mRNA translational efficiency [14-18]. In addition to ICU, a significant influence of codon pair usage, also known as Codon Context (CC), at the level of gene expression has been reported in several studies and is suggested to be a result of potential tRNA-tRNA steric interaction within the ribosome [18]. For that reason, the CC was also incorporated into current gene design tools [18,19].

It is important to note that the codon usage optimization may not need to concern the whole gene to result in substantially increased gene expression. There is evidence suggesting that the initial 15–25 codons of the Open Reading Frame (ORF) deserve special consideration [11]. It was shown that the impact of rare codons on translation rate is particularly strong in these first codons for expression in both *E. coli* and *Saccharomyces cerevisae*[11]. This phenomenon is even more profound for the initiation codon. For example, replacing the native TTG initiation codon with an ATG codon resulted in high-level expression of the previously silent *bspRIR* gene in *E. coli*, which encodes BspRI REase [20].

Other known strategies for the improvement of recombinant gene expression include: (*i*) avoiding secondary mRNA structures in gene design; (*ii*) displacing mRNA structure from the initiation region or improving the physical integrity of the protein by the addition of N-terminal fusion tags [11]; and (*iii*) targeted and global bacterial genetic/strain engineering to enhance recombinant protein production [21].

Investigating members of the *Thermus sp.* enzyme family of atypical bifunctional REases-MTases that we previously described [22-29], we encountered serious difficulties concerning low expression levels of these thermophile-derived genes in *E. coli*. Thus far we have successfully cloned and expressed six thermophilic genes from the family [26,27,29], *this work*], coding for the related thermostable enzymes: TspGWI [22,25,26], TspDTI [24,27], Tth111II/TthHB27I [27], *unpublished results*], TsoI [27,29] and TaqII [23,24,28]. Moreover, according to the recent bioinformatic analyses and literature data, we predicted the existence of putative or partially analysed members (or genes) related to the *Thermus* sp. family originating from evolutionary distant mesophilic bacteria [29]. All members of the family are sub-Type IIS/IIG/IIC REases. They recognize asymmetric DNA sequences, cleave 11/9 nt downstream, possess REase and MTase activities within the same polypeptide and their REase activity is affected by SAM or its analogues [22-29]. Bioinformatic analyses coupled with site-directed mutagenesis experiments defined distinct functional regions, fused within a single polypeptide: a tandemly arranged Type I-like domains, a central HsdM-like module (helical domain), a conserved MTase domain and an N-terminal nuclease domain, similar to the corresponding domains in HsdR subunits [26,27]. These data indicate that, structurally and functionally, the *Thermus sp*. enzyme protomers correspond to the streamlined ‘half’ of a Type-I enzyme [26,27].

In this study we describe a successful strategy for cloning and expression of a ‘toxic’, fully synthetic *taqIIRM* gene, designed for a significant improvement of biologically active recombinant prototype TaqII REase-MTase production in *E. coli.* Using the ‘one amino acid–one codon’ strategy, we intentionally avoided excessively high expression, which would be detrimental to recombinant cells, due to the protein’s high ‘toxicity’. This variant of the ‘one amino acid–one codon’ strategy is biased towards a low AT content and is suitable for other thermostable REases. We also anticipate its usefulness for non-REase-related genes, originating from thermophiles, including those coding for industrial enzymes.

## Results and discussion

### Design and cloning of a synthetic *taqIIRM* gene and comparison to wt *taqIIRM* gene from *Thermus aquaticus* (*T. aquaticus*)

**Figure 1:**
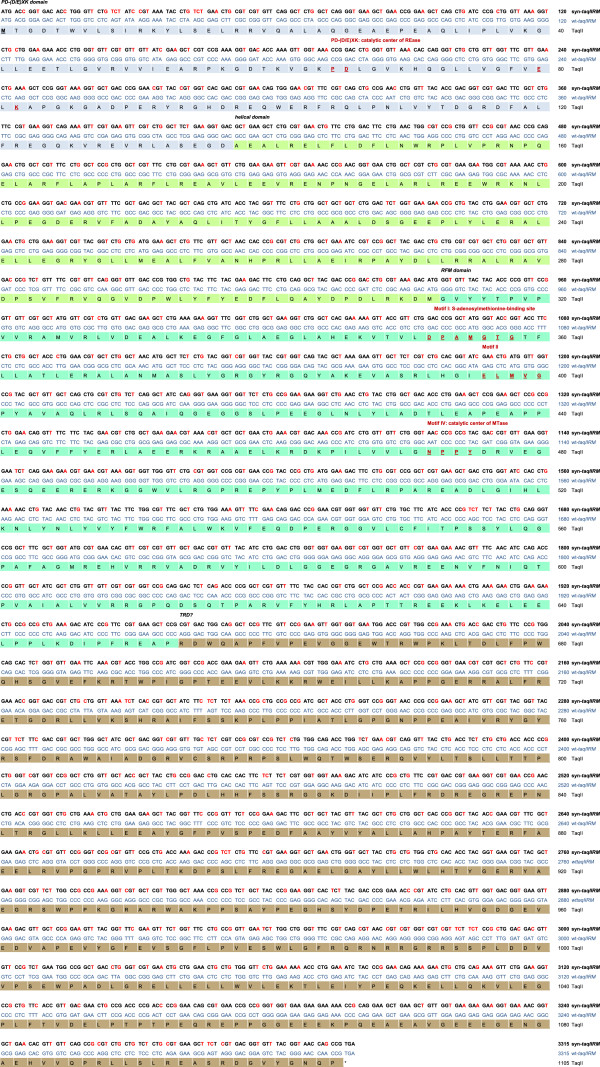
**Differences in DNA sequences of the synthetic and wt recombinant *taqIIRM *genes.** The predicted aa sequence of the 125.7 kDa TaqII protein is indicated in capital letters. The DNA sequence of the wt-*taqIIRM* gene is indicated in blue italics. The DNA sequence of the syn-*taqIIRM* gene is shown in black bold letters and the changed bases are marked in red. The crucial amino acids of the catalytic centres are dark red, bold and underlined. The functional protein domains are marked as follows: REase domain in blue, helical domain in light green, MTase domain in dark green and the potential TRD region in brown. Numbering of nt of *taqIIRM* gene variants and polypeptide aa starts as ‘1’ with the beginning (ATG) of *taqIIRM* ORF.

**Figure 2:**
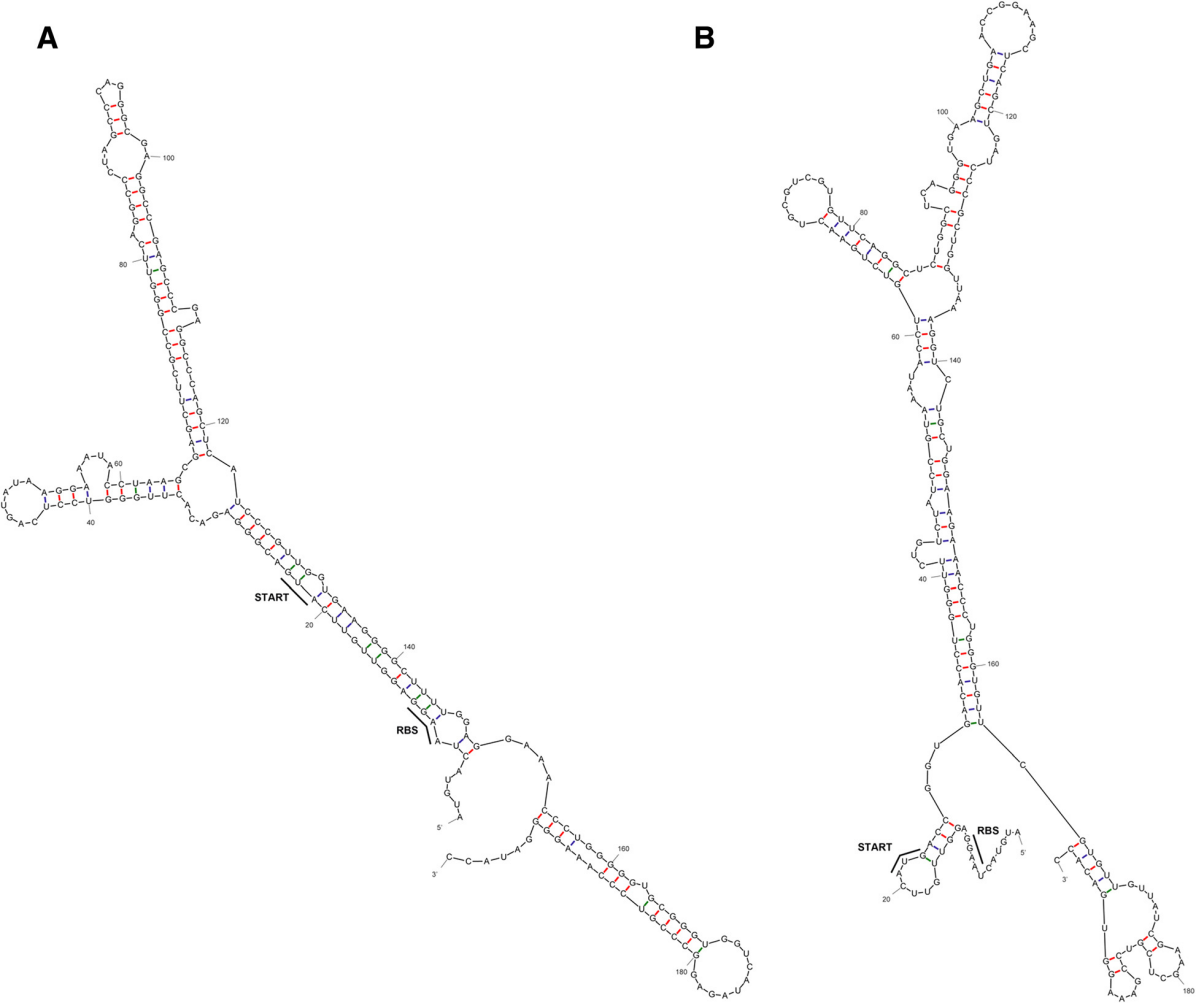
**Secondary structure of the first 200 nt of *taqIIRM *mRNA generated by Mfold Web Server **[31,32]**. (A)** Structure of initial *taqIIRM* mRNA fragment before codon optimization (revised free energy: dG = −84.5 kcal/mol). **(B)** Structure of initial *taqIIRM* mRNA fragment after codon optimization (revised free energy: dG = −63.33 kcal/mol).

The *taqIIRM* gene was sequenced *de novo* by a combination of PCR products, obtained using the *T. aquaticus* genomic template, a proofreading DNA polymerase and direct genomic dideoxy and NGS sequencing approaches. The obtained extended sequence contig contained previously published *taqIIRM* gene sequence data (without expression analysis) [GenBank: AY057443, AAL23675.1] [30], with an error corrected, located outside the *taqIIRM* ORF, coding for a 125.7 protein. Furthermore, the gene is preceded by a sub-optimal ribosome-binding-site 5′-GGAG-3′, located 6 bp upstream of the ORF start codon [GenBank: KF92665]. Subsequently, the wt gene was converted to a novel artificial gene, which radically departs from the wt *taqIIRM* nucleotide sequence, while maintaining the same aa sequence (Figure 1) [GenBank: KF894945]. Here we show the designing of a synthetic 3315 bp *taqIIRM* gene (syn-*taqIIRM*), cloning, expression and isolation of the recombinant enzyme. A total of 718 out of 1105 codons were changed, thus comprising a massive 65% portion of the ORF. For comparative purposes, we also cloned *de novo* and expressed the wt gene (wt-*taqIIRM*), PCR amplified from *T. aquaticus* genomic DNA. Analysis of the wt-*taqIIRM* gene (66.3% GC) [GenBank: KF92665] revealed that at least 56.4% of codons are not the preferred for highly expressed *E. coli* genes (Table 1). Due to the previously observed low expression of the *Thermus* sp. family genes in *E. coli*[26,27], we assumed that the codon optimization coupled with mRNA secondary structure reduction and a generally decreased GC content of the *taqIIRM* gene, leading to relaxing of the DNA-RNA duplexes and RNA-RNA secondary structures, might result in an increase of TaqII protein synthesis. Therefore, a synthetic variant of the *taqIIRM* gene (with only 76.5% nt sequence identity to the wt gene) was designed using a modified ‘one amino acid–one codon’ method [GenBank: KF894945] [11,13]. Figure 1 shows wt-TaqII and syn-TaqII nt sequences as well as functional domains and motifs that we have previously determined by bioinformatics analysis [26] and further confirmed experimentally [manuscript in preparation]. Consequently, bioinformatic prediction of secondary structures (Mfold Web Server [31,32]) of the first 200 nt of mRNA’s, coding for wt-*taqIIRM* and syn-*taqIIRM* genes (Figure 2), has revealed that the ATG start codon and RBS are much more exposed in mRNA transcribed from the optimized gene (Figure 2B) than from the wt gene (Figure 2A). In wt mRNA the translation signals are hidden in a double stranded (ds) RNA helix with substantial stability (revised free energy: dG = −84.5 kcal/mol). On the contrary, ATG and RBS of syn-*taqIIRM* mRNA are located on a single-stranded (ss) region and the mRNA ds structure has substantially higher flexibility, as it exhibits revised free energy dG = −63.33 kcal/mol.

**Table 1:** **Codon distribution of wt and synthetic *taqIIRM *sequences**

**aa**	**Codon**	**Fraction in *E. coli***^**1**^	**WT**	**SYN**	**aa**	**Codon**	**Fraction in *E. coli***^**1**^	**WT**	**SYN**
**Ala (A)**	**GCU**	0.35	5	93	**Leu (L)**	**CUG**	0.83	52	145
	GCA	0.28	2	0		CUC	0.07	63	0
	GCG	0.28	24	0		CUU	0.04	11	0
	GCC	0.10	62	0		UUG	0.03	9	0
						UUA	0.02	2	0
						CUA	0.00	8	0
**Arg (R)**	**CGU**	0.74	7	108	**Lys (K)**	**AAA**	0.74	11	39
	CGC	0.25	29	0		AAG	0.26	28	0
	CGA	0.01	1	0					
	AGG	0.00	31	0					
	AGA	0.00	3	0					
	CGG	0.00	37	0					
**Asn (N)**	**AAC**	0.94	17	19	**Met (M)**	**ATG**	1	9	9
	AAU	0.06	2						
**Asp (D)**	**GAC**	0.67	41	49	**Phe (F)**	**UUC**	0.76	35	43
	GAU	0.33	8	0		UUU	0.24	8	0
**Cys (C)**	**UGC**	0.51	1	2	**Pro (P)**	**CCG**	0.77	14	94
	UGU	0.49	1	0		CCA	0.15	4	0
						CCU	0.08	14	0
						CCC	0.00	62	0
**Gln (Q)**	**CAG**	0.86	27	33	**Ser (S)**	***UCC***	0.37	14	0
	CAA	0.14	6	0		**UCU**	0.34	0	36
						AGC	0.20	12	0
						UCG	0.04	4	0
						AGU	0.03	6	0
						UCA	0.02	0	0
**Glu (E)**	**GAA**	0.78	33	122	**Thr (T)**	**ACC**	0.55	24	37
	GAG	0.22	89	0		ACU	0.35	4	0
						ACG	0.07	7	0
						ACA	0.04	2	0
**Gly (G)**	**GGU**	0.59	4	91	**Trp (W)**	**UGG**	1	23	23
	GGC	0.39	33	0					
	GGG	0.02	37	0					
	GGA	0.00	17	0					
**His (H)**	**CAC**	0.83	16	17	**Tyr (Y)**	**UAC**	0.75	43	43
	CAT	0.17	1	0		UAU	0.25	0	0
**Ile (I)**	**AUC**	0.83	20	24	**Val (V)**	**GUU**	0.51	5	78
	AUU	0.17	0	0		GUA	0.26	8	0
	AUA	0.00	4	0		GUG	0.16	31	0
						GUC	0.07	34	0

^1^Fraction of relative occurrences of the codon in its synonymous codon family [33]. Bold underlined – codons selected for syn-TaqII construction. Bold italics underlined – Ser codon, which is the most frequently used in *E. coli*.

For the ‘one amino acid-one codon’ approach, the most preferred codon in the highly expressed *E. coli* genes was selected for every aa (Table 1; Figure 1). A single exception was made in the case of the serine codon: from two nearly identically frequent codons, UCC and UCU, the latter was selected as it has a lower GC content, even though it is used at slightly lower rate as UCC in highly expressed *E. coli* genes (Table 1; Figure 1). It was hypothesized that such an approach might result in a lower level of expression than the maximum obtainable with the use of the set of most frequent codons, specific for each as a random, weighted mixture. It was shown experimentally that a ‘codon randomization’ method approach leads to higher gene expression by preventing depletion of the aminoacyl-tRNAs pool and consequently slowing down translation, stalling ribosomes or prematurely terminating translation [11,13]. As codons are assigned randomly, this method allows for the generation of countless gene variants [13]. This allows for further nt sequence fine-tuning, without altering the final aa sequence. Thus, further removal of mRNA secondary structures, considering ICU, CC factors is possible.

However, sub-optimal gene optimization, using the ‘one amino acid-one codon’ strategy over the ‘codon randomization’ strategy, may be beneficial in some cases by reducing metabolic stress imposed on the recombinant host, which has to repair cellular damages caused by overproduction of ‘toxic’ heterologous proteins. Excessive expression of such proteins would result in poor recombinant host growth, activity-less mutations appearing in the cloned gene and a natural selection for mutant-carrying bacteria during cultivation, cell fragility and spontaneous lysis, among others. Another, more subtle effect might be associated with co-translational folding, where the availability of isoacceptor tRNA molecules regulates folding kinetics. Thus, the obtained expressed proteins may vary in properties, depending on whether they were synthesised basing on the fastest possible translation constructs or moderately boosted genes. TaqII, originating from a thermophile, is very large for a Prokaryotic protein (125.7 kDa) and contains functional (and perhaps physical) domains. For that reason folding kinetics may play a role in the final active state of the recombinant protein variants. As a result of the factors listed above, the final recombinant protein yield for production purposes may actually be lower and less predictable with the use of maximum expression constructs, than while using moderately expression-boosted, but stable, recombinant constructs. Thus, our motivation behind using the ‘one amino acid-one codon’ strategy for the syn-*taqIIRM* gene construction was to stabilize recombinant constructs by preventing excessively high expression of the TaqII REase-coding gene, ‘toxic’ for a bacterial host. To reduce *taqIIRM* gene ‘toxicity’, we used a strictly controlled λ P_R_ promoter and a very low permissive cultivation temperature of 28°C, which not only kept the λ P_R_ promoter silent, but also further decreased the activity of any thermostable TaqII molecules, originating from residual expression under permissive conditions. Despite strict promoter control we still observed increased fragility of recombinant *E. coli* cells, expressing the *taqIIRM* gene. This is a general phenomenon, which we have also observed in the case of other cloned, *Thermus* sp. family REases.

**Figure 3:**
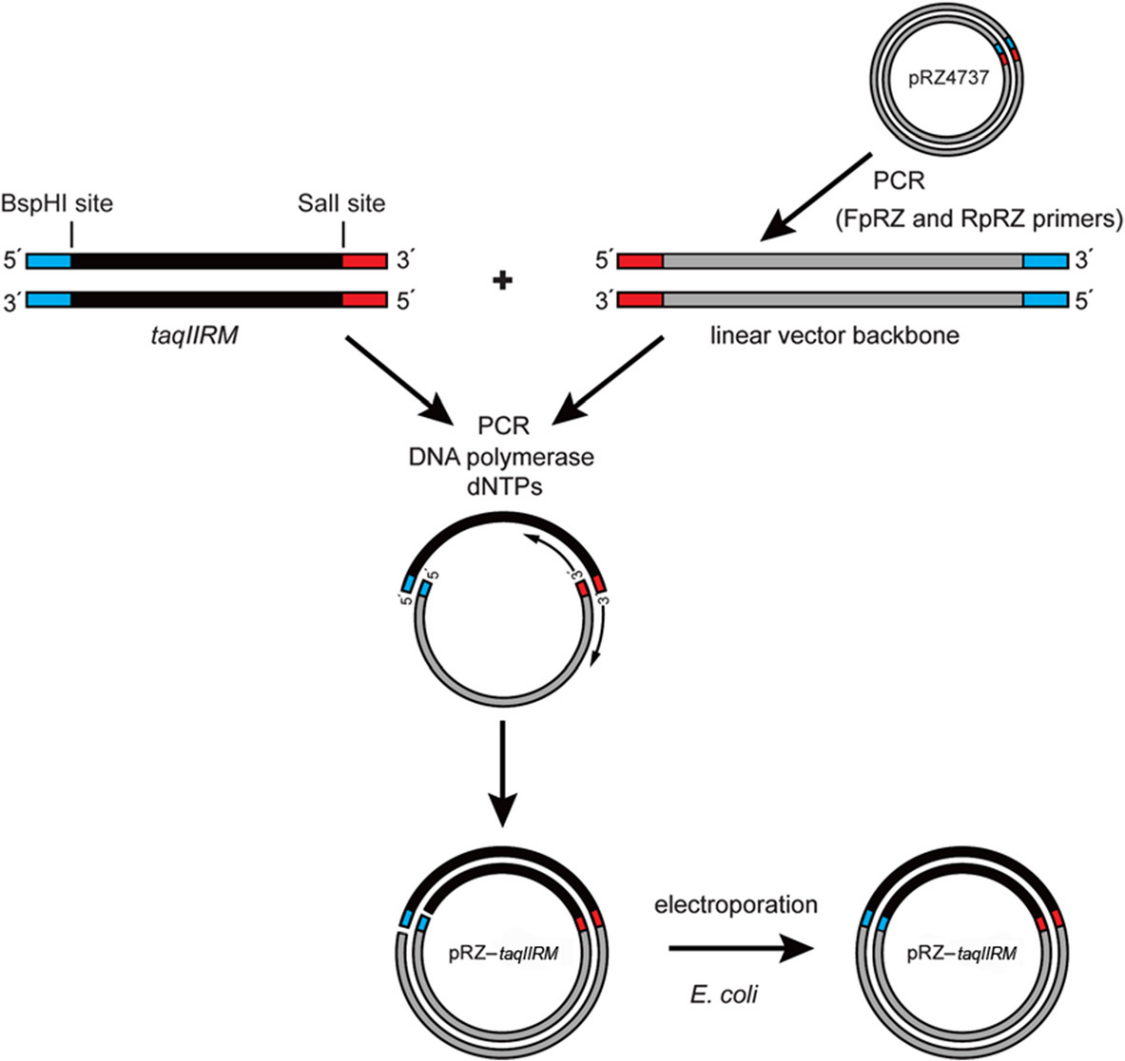
**Scheme of the *taqIIRM *gene cloning method.** The *taqIIRM* gene variants (shown in black) were amplified using a PCR (see Methods section). DNA assembly and circularization was performed. The corresponding head and tail sequences of the vector (shown in grey) and gene were annealed and assembled. For simplicity only one possible variant of the DNA assembly was shown. The complementary ends of the primers are in red or blue. The final DNA constructs (pRZ-*taqIIRM*) were used to transform *E. coli* cells.

The codon-optimized synthetic gene was generated by a commercial service using ss 5′-phosphorylated, overlapping complementary primers, subjected to ligation. Finally, the fully assembled gene was amplified with a proofreading DNA polymerase. The resulting synthetic gene (55.9% GC) was further enriched with two DNA fragments, overlapping the sequence of a modified pRZ4737 vector DNA (Table 2; sequence written in small letters). For that purpose, two oligodeoxyrybonucleotides (oligos) were used (Table 2) and an additional PCR reaction with a proofreading DNA polymerase was performed (see Methods section). Finally, the gene was assembled with the complementary modified pRZ4737 vector linear backbone, with gene expression driven by a λ P_R_ promoter, inducible by a temperature shift to 42°C. The DNA assembly was performed using a ‘one-step DNA fragment assembly and circularization’ method, without DNA ligation needed [34] (Figure 3). The expression temperature of 42°C was selected to ensure adequate folding of the thermostable TaqII protein. As a control, a wt *taqIIRM* gene was cloned to the modified pRZ4737 using the same cloning strategy (see Methods section).

**Table 2:** **DNA sequence of PCR primers used for wt-*taqIIRM *and syn*-taqIIRM *genes cloning**

**Name**	**DNA sequence**	**Target**
**FsynTaq**	5′-**tgataatggttgcatgtactaaggagg**ttgttcATGACCGGTGACACCTGGGTTCTGT-3′	syn*-taqIIRM* gene
**RsynTaq**	5′**-acacaggaaacagaccatggaagtcga*c****TA*CGGCTGGTTACCGTAAACACCGTCAC-3′	
**Ftaq**	5′-**tgataatggttgcatgtactaaggagg**ttgttcATGACCGGAGACACTTGGGTCCTCA-3′	wt-*taqIIRM* gene
**Rtaq**	5′**-acacaggaaacagaccatggaagtcgac***TCA*CGGTTGGTTCCCGTAGACTCCGTCC-3′	
**FpRZ**	5′-**gtcgacttccatggtctgtttcctgtgt**-3′	Linear pRZ4737 vector backbone
**RpRZ**	5′-**cctccttagtacatgcaaccattatca**-3′	

The introduced BspHI and SalI restriction sites are underlined. DNA fragments complementary to the modified pRZ4737 vector are written in small letters. DNA fragments complementary to the wt-*taqIIRM* and syn*-taqIIRM* genes are in capital letters. Stop codons are in italics. The corresponding complementary regions of forward and reverse primers are marked in bold.

### Improved expression of the thermophile-based synthetic recombinant *taqIIRM* gene in mesophilic *E. coli*

**Figure 4:**
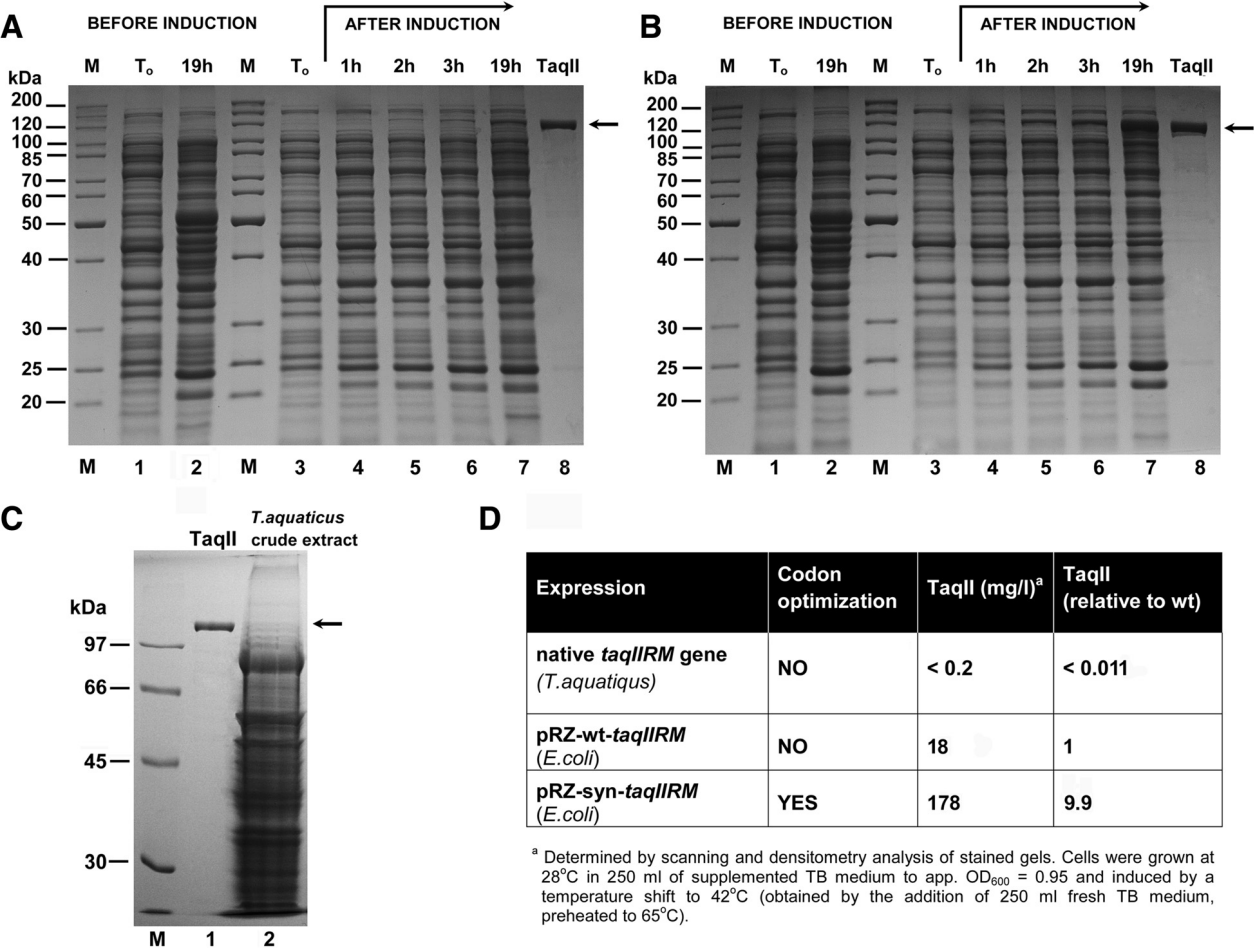
**Expression of the synthetic and wt recombinant *taqIIRM *gene variants. (A)** Expression of the wt- recombinant *taqIIRM* gene. Lanes M, protein marker (Thermo Fisher Scientific/Fermentas); lane 1, control culture – crude lysate from *E. coli* expressing the cloned wt-*taqIIRM* gene, without induction (OD_600_ = 0.95); lane 2, control culture after 19 h of cultivation; lane 3, crude lysate from *E. coli* expressing the cloned wt-*taqIIRM* gene, before induction (OD_600_ = 0.9); lane 4, 1 h after induction; lane 5, 2 h; lane 6, 3 h; lane 7, 19 h; lane 8, purified, homogeneous recombinant syn-TaqII protein. **(B)** Expression of the recombinant syn-*taqIIRM* gene. Lanes M, protein marker (Thermo Fisher Scientific/Fermentas); lane 1, control culture – crude lysate from *E. coli* expressing the cloned syn-*taqIIRM* gene, without induction (OD_600_ = 0.95); lane 2, control culture after 19 h of cultivation; lane 3, crude lysate from *E. coli* expressing the cloned syn-*taqIIRM* gene, before induction (OD_600_ = 0.95); lane 4, 1 h after induction; lane 5, 2 h; lane 6, 3 h; lane 7, 19 h; lane 8, purified, homogeneous recombinant syn-TaqII protein. **(C)** Expression of the native *taqIIRM* gene in *T. aquatiqus*. Lane M, protein marker (GE Healthcare, Little Chalfont, United Kingdom); lane 1, purified, homogeneous recombinant syn-TaqII protein; lane 2, crude lysate from *T. aquatiqus*. **(D)** The amount of TaqII protein produced by *E. coli* cells expressing *taqIIRM* gene variants versus protein yield from native source.

**Figure 5:**
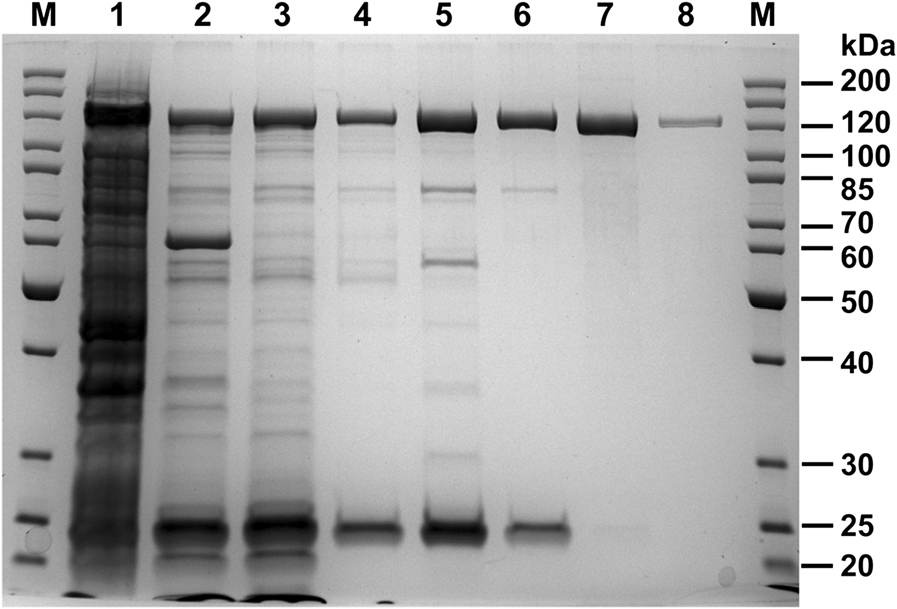
**Isolation of recombinant syn-TaqII protein from *E. coli*.** Lane M, protein marker (Thermo Fisher Scientific/Fermentas); lane 1, crude lysate; lane 2, supernatant after incubation at 65°C; lane 3, supernatant after PEI; lane 4, 30-50% ammonium sulphate (AmS) fractionation; lane 5, P11 chromatography; lane 6, DEAE chromatography; lane 7, size exclusion chromatography; lane 8, recombinant wt-TaqII protein.

Similar to other genes from the investigated *Thermus sp*. family, low expression of the native *taqIIRM* gene in the *T. aquaticus* results in a very small yield of active TaqII protein (lower than 0.2 mg/L culture) (Figure 4C,D). Moreover, the native TaqII protein isolation from *T. aquaticus* is impaired by the presence of vast amounts of non-specific nucleases and another REase - TaqI - as well as abundant amounts of pigments and other cellular components, which strongly interfere with chromatographic separations and enzymatic assays [35]. To improve the expression of the gene and to increase the protein yield, two *taqIIRM* gene variants (wt and synthetic) were cloned and expressed in *E. coli* (Figure 4A,B,D; Figure 5). Initial wt *taqIIRM* cloning [Genbank: AY057443, AAL23675.1] was conducted using a different strategy than presented in this paper and is to be published elsewhere. The amount of TaqII protein produced by the expression of each gene variant was quantified by densitometry of the stained SDS/PAGE gels and is shown in Figure 4D. Consistent with the results obtained from gel scanning quantification, the yields of protein for the synthetic and wt gene were 178 mg/l and 18 mg/l, respectively, thus reaching on average app.10-fold expression increase. We have obtained such expression levels in several experiments. The TaqII protein yields are relatively high, even though it is a ‘toxic’ protein. However, being a thermostable enzyme, it exhibits decreased activity at lower temperatures used for recombinant *E. coli* cultivation. The high TaqII yields are also attributed to the development of a rapid and efficient purification protocol as well as to the bacteria cultivation conditions, which include overnight growth with vigorous aeration after induction. As a result, high cell densities are obtained, leading to an increased bacterial mass per litre of culture. The presence of recombinant TaqII both in entire cells and in the soluble fraction was confirmed using enzymatic activity assays as well as SDS/PAGE and has shown that the enzyme is fully soluble (not shown). The high expression boost findings are in contrast to the report [13] that showed a relatively small expression increase with the use of the ‘one amino acid-one codon’ gene optimization method, explained by depletion of the tRNAs variants, assigned for single codon types. Moreover, such cell deprivation also induces translation errors, thus decreasing protein-specific activity. Here we show that the ‘one amino acid-one codon’ combined with weighting toward low GC content codons (in this case, serine codons), allows for a significant expression increase of a thermophile gene in the recombinant host. Even though no comparison was made between the two equivalent variants (using alternatively UCC or UCU serine codons) of the synthetic gene, we hyphothesize that the achieved high expression points to the fact that using less frequent codons, but with a lower AT content is not detrimental to the high expression of a synthetic gene. Thus, modifications of this method, namely further biasing towards other aa variants with similar codon usage as most frequently used codons, may be an interesting avenue for future exploration. Besides codon optimization, the GC content was significantly decreased by 10.4%. Any further GC content decrease was limited by the aa sequence of the TaqII protein. Together with the post-optimization sequence scanning for mRNA secondary structures (Figure 2), codon clusters and the local codon environment, the final synthetic gene has become ‘*E. coli* friendly’ with the preferred codons content and ATG start codon as well as RBS exposed in a ss mRNA segment, allowing for a one-order of magnitude increase in *taqIIRM* expression, as detected by the cellular protein enzymatic assays and SDS/PAGE. The method was devised for ‘toxic’ REase-coding genes in particular – however, it seems well suited for general industrial thermostable enzyme production, including those ‘toxic’ to their recombinant hosts *via* different mechanisms than REases. As expression results reported in literature vary greatly for different genes being optimized, the issue is complicated and, apparently, multiple factors, not always defined, affect the final protein yield outcome. Our results are meant to be an experimental data contribution to the discussion, which may become useful to solve thermophile gene-derived expression problems. Besides the anticipated, more general usefulness of the modified, AT-content biased gene design method, the major novelty of the presented work is also attributed with the optimization target chosen - the sub-Type IIS/IIC/IIG TaqII thermostable REase. The enzyme is a new tool for DNA manipulation purposes, as it exhibits a prototype DNA-cleavage specificity. We present for the first time the *taqIIRM* cloning method, as only the wt *taqIIRM* nt sequence has been previously deposited in GenBank [30]. Moreover, we have recently published [28] a new method for quasi-random genomic libraries generation, by the development of chemically-induced TaqII REase specificity relaxation from 6-bp to a combined 2.9-bp cognate site. This was achieved by including the enzyme’s cofactor analogue into the DNA digestion reaction. Thus, we anticipate an increased interest in practical usage of the enzyme in DNA cloning technologies.

### Enzymatic properties of recombinant TaqII enzyme variants

The recombinant TaqII protein (syn-TaqII), isolated from the recombinant *E. coli* strain harbouring the pRZ-syn-*taqIIRM* expression plasmid (Figure 5, lane 7), was used for the study of the enzyme biochemical properties, reaction conditions, cofactors and their analogues that influence DNA cleavage and/or the methylation activity. The purification scheme included mid-scale isolation, app. 50 g cells, which were suspended in a buffer with pH and salt concentrations stabilizing the enzyme (not shown). In addition, glycerol and non-ionic detergents were added to block hydrophobic patches on the TaqII protein surface and prevent the protein from denaturation, aggregation and adhesion. After ultrasonic disruption and centrifugation of cell debris, the crude lysate was subjected to a heating step at 65°C (Figure 5, lane 2). This stage was critical to remove most mesophilic *E. coli* host proteins and inactivate non-specific nucleases. The thermal inactivation step was important to obtain a DNA degradation-free purified enzyme preparation, thus suitable for practical applications in molecular cloning methodology as a new prototype specificity. Further precipitation steps included polyethyleneimine (PEI) removal of nucleic acids and residual acidic proteins (Figure 5, lane 3), followed by fractionated precipitation with AmS (Figure 5, lane 4). The above three precipitation methods used, each based on a different principle, were sufficient to obtain an enzyme yielding high quality DNA digests, although it was not a homogeneous protein (Figure 5, lane 4). Further purification included ion exchange on cationite phosphocellulose P11 (Figure 5, lane 5), which also served as a semi-affinity medium, due to the presence of phosphate groups, followed by anionite ion exchange on DEAE-cellulose (Figure 5, lane 6). The nearly homogeneous preparation was then subjected to molecular sieving to remove any trace contaminants, taking advantage of the high molecular weight of TaqII (Figure 5, lane 7).

Both recombinant TaqII protein variants were also subjected to analytical molecular sieving in a buffer with a composition close to the physiological conditions containing 3 mM MgCl_2_ (in the absence of DNA). The experiment revealed that the molecular size of both variants is in the range 110–130 kDa, indicating that under physiological conditions, the proteins exist as monomers, identical to the previously described native enzyme [24]. Moreover, the apparent molecular size of the recombinant protein variants under denaturing conditions was found to be sligthly over 120 kDa, very similar to the molecular mass of TaqII isolated from *T. aquaticus*, which was analysed with the use of different molecular size markers [24].

**Figure 6:**
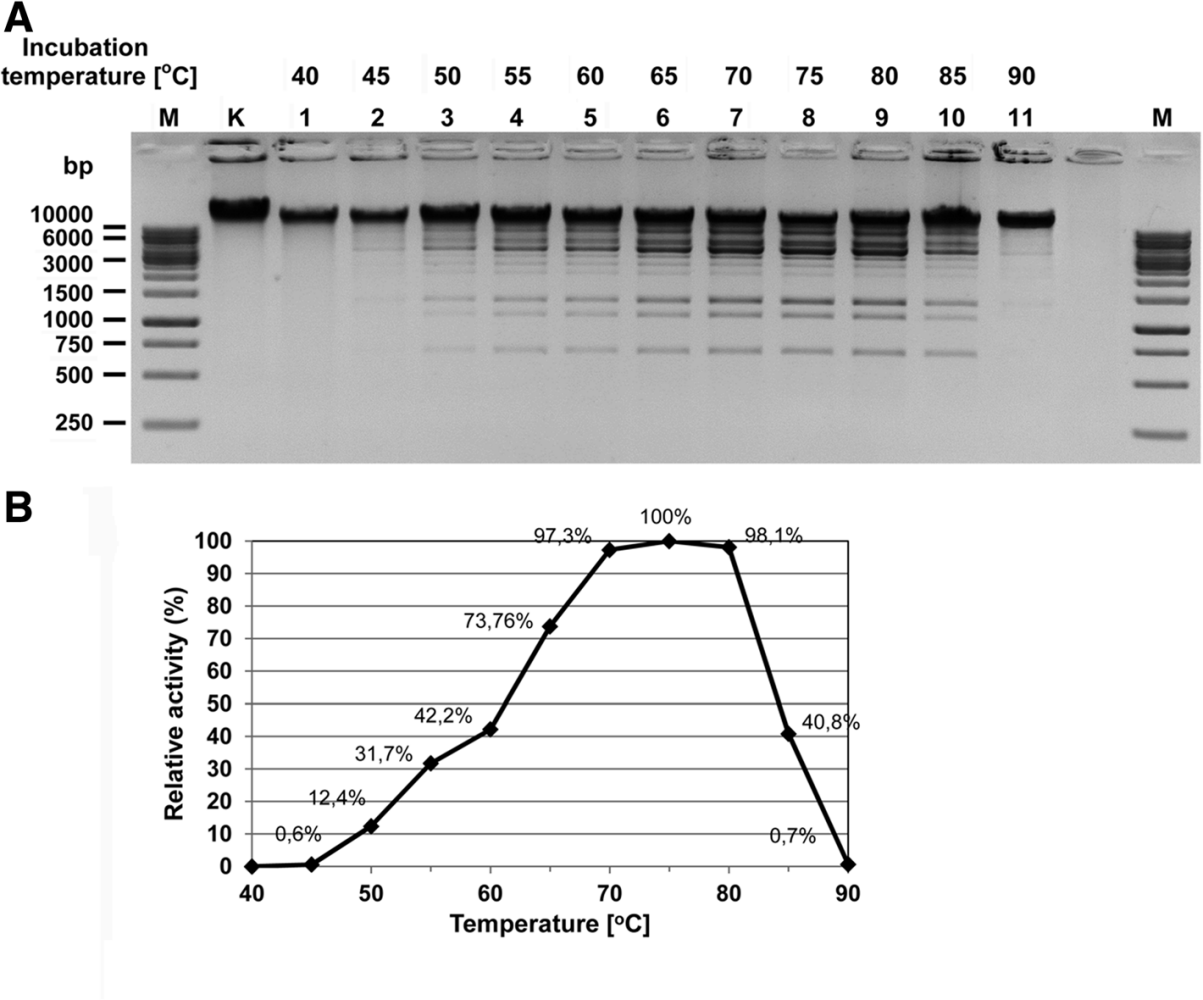
**Temperature range of the recombinant TaqII REase activity. (A)** The temperature range of syn-TaqII REase enzymatic activity. 1 μg of bacteriophage λ DNA (= 0.32 pmol recognition sites) was digested with 23 pmol (0.16 u) of syn-TaqII in the optimal buffer supplemented with 100 μM SIN for 1 h in the temperature range from 40 to 90°C. Lane M, GeneRuler™ 1 kb DNA Ladder (Thermo Fisher Scientific/Fermentas); selected bands marked); lane K, undigested λ DNA; lane 1, 40°C, lane 2; 45°C; lane 3, 50°C; lane 4, 55°C; lane 5, 60°C; lane 6, 65°C; lane 7, 70°C; lane 8, 75°C, lane 9, 80°C; lane 10, 85°C, lane 11, 90°C. **(B)** The dependence of relative activity of syn-TaqII on the reaction temperature.

As expected, the recombinant TaqII maintains the absolute requirement for Mg^2+^ for cleavage activity. The temperature activity range of the recombinant TaqII REase extends from 40°C to 85°C, with the maximum observed at 70–80°C (Figure 6). Remarkably, the upper activity limit extends well beyond the *T. aquaticus* growth range by approximately 10°C. This indicates that different cellular components are becoming limiting factors for cell survival at different temperatures, thus no simple ‘thermostability’ explanation can be given in a thermophile characterization. Those findings are in contrast to our previous observations regarding another member of the *Thermus* sp. family – TsoI, exhibits remarkably lower thermostability, by app. 10-15°C than optimum growth temperature of TsoI-coding *Thermus scotoductus* bacteria. As RM systems exhibit a tendency towards horizontal transfers between species, a higher than expected temperature maximum of TaqII and lower than expected temperature maximum of TsoI may indicate that these enzymes have been acquired in the past from more thermophilic or more mesophilic bacteria, respectively. Finally, such high TaqII thermostability may be of practical use in DNA manipulation methodologies. Incubation at 37°C resulted in no detectable REase activity under our assay conditions (data not shown). Recombinant TaqII is inactivated at temperatures above 90°C.

**Figure 7:**
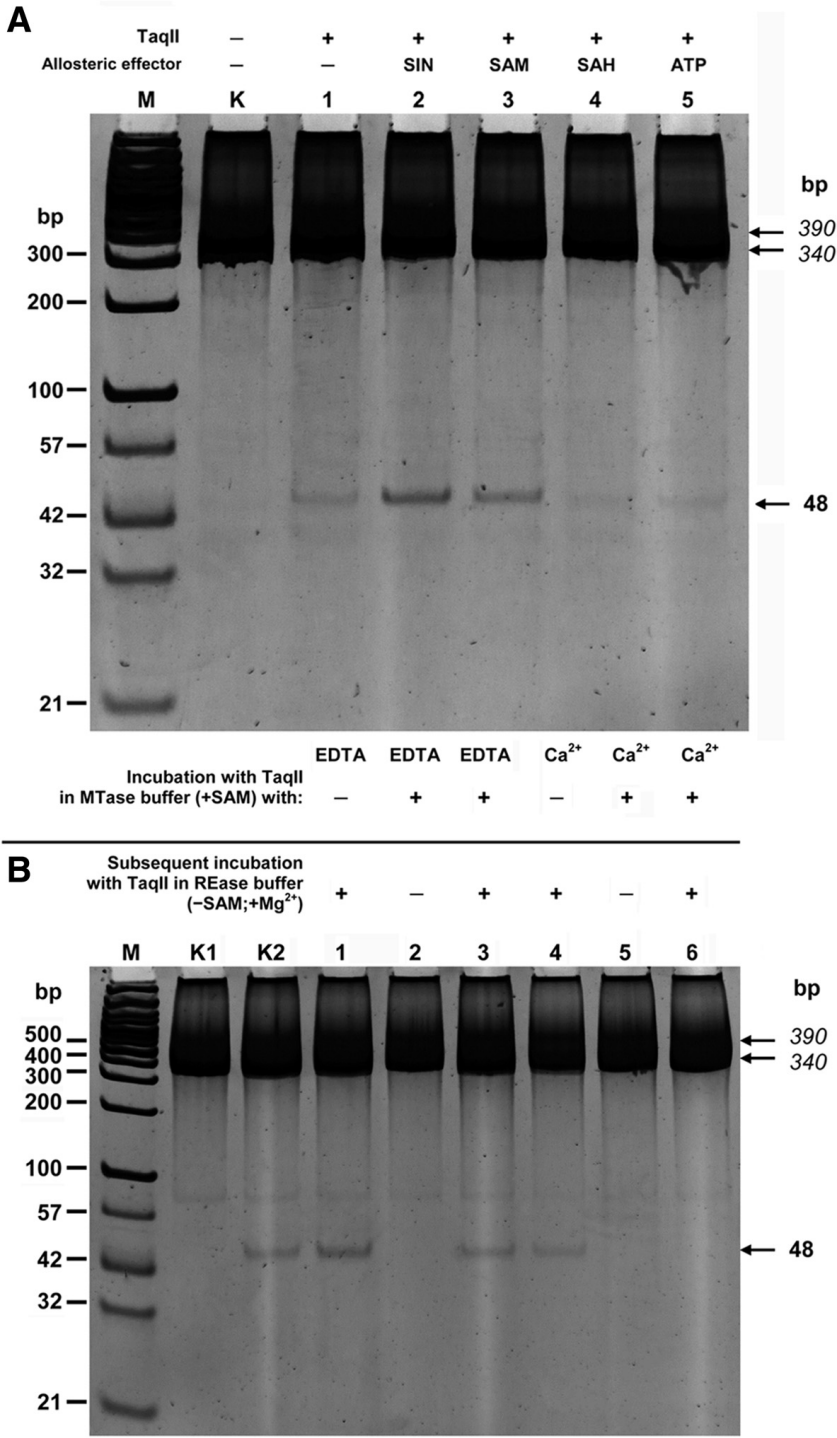
**Bifunctionality of TaqII: REase/MTase activities of the enzyme. (A)** Evaluation of cofactor SAM effect and its analogues on TaqII activity. Three putative effectors and ATP, were compared in their effect on syn-TaqII REaseactivity. 300 ng of the PCR fragment (390 bp; = 1.2-pmol of 5′-GACCGA-3′ recognition sites) was digested with 17 pmol (0.12 u) of syn-TaqII as described in Methods. Lanes M, modified GeneRuler™ 100 bp DNA Ladder (Thermo Fisher Scientific/Fermentas); lane K, untreated DNA; lane 1, + syn-TaqII (no cofactors, except Mg^2+^); lane 2, as in lane 1 + SIN); lane 3, + SAM; lane 4, + SAH; lane 5, + ATP. DNA was treated with limited amounts of syn-TaqII, to pinpoint stimulatory effect differences. **(B)** The MTase activity of syn-TaqII. Samples of 1.2 pmol 390 bp PCR fragment were incubated with 30 pmol syn-TaqII protein in the MTase buffer in the presence of either EDTA or Ca^2+^ as described in Methods. The resulting DNA was purified and challenged with an excess of TaqII REase: 1.18 pmoles the enzyme and 0.6 pmoles 5′-GACCGA-3′ sites (2 : 1 molar ratio) for 1 h at 65°C in the optimal TaqII REase buffer supplemented with 10 mM MgCl_2_; Lane M, as in panel **A**; lane K1, untreated DNA; lane K2, no TaqII/ REase buffer; lane 1, + TaqII, MTase buffer + EDTA/subsequent incubation + TaqII, REase buffer; lane 2, + TaqII, MTase buffer + EDTA/no subsequent incubation; lane 3, + TaqII, MTase buffer + EDTA/subsequent incubation with TaqII, REase buffer; lane 4, no TaqII, MTase buffer + Ca^2+^/subsequent incubation + TaqII, REase buffer; lane 5, + TaqII, MTase buffer + Ca^2+^/no subsequent incubation; lane 6, + TaqII, MTase buffer + Ca^2+^/subsequent incubation + TaqII, REase buffer.

Similar to other bifunctional Type-IIC/IIG REases-MTases [36,37], both SAM (Figure 7A, lane 3) and its analogue SIN (Figure 7A, lane 2) stimulate the recombinant syn-TaqII REase activity, while DNA methylation reaction by-product S-adenosylhomocysteine (AdoHcy) and ATP have no effect on DNA cleavage (Figure 7A, lanes 4 and 5). Similar to the previously investigated members of the *Thermus sp*. enzyme family [22-27,29], the recombinant TaqII protein exhibits specific, cognate MTase activity (Figure 7B), which is highly stimulated by the presence of either Ca^2+^ (Figure 7B, lane 6) or Mg^2+^ ions (data not shown). Thus, both recombinant variants of TaqII REase-MTase exhibit the same enzymatic characteristics in the assays performed here.

## Conclusions

The novelty of the presented work includes:

i. Design of entirely synthetic, low GC content and mRNA secondary structures, long 3315 bp *taqIIRM* gene with optimized codons to enhance its expression in *E. coli*;

ii. Cloning of the sub-Type IIS/IIC/IIG thermostable REase syn-TaqII - a new tool for DNA manipulation purposes, which includes the use of TaqII prototype REase specificity for DNA cleavage as well as for specialized applications in quasi-random genomic libraries generation [28];

iii. Expression of optimized synthetic *taqIIRM* gene in *E. coli* under the control of **λ***P*_*R*_ promoter that has resulted in an approximately 10-fold increase as compared to the cloned, native *taqIIRM* gene;

iv. Development of rapid and efficient TaqII purification protocols and the recombinant enzyme’s characterization;

v. Displaying evidence that in contrast to other reports [13], the modified ‘one amino acid–one codon’ method allows for a significant increase of REase-coding gene expression in recombinant *E. coli*, which can be suited more generally for the industrial production of other thermostable enzymes.

## Methods

### Bacterial strains, plasmids, media and reagents

*T. aquaticus* YT was obtained from American Type Culture Collection. *E. coli* DH5α {F– Φ80∆*lacZ*∆M15 ∆ (*lac*ZYA*-arg*F) U169 *rec*A1 *end*A1 *hsd*R17(r_K-_, m_K+_) *pho*A *sup*E44 λ*-thi-1, gyr*A96*, rel*A1} (Life Technologies, Gaithersburg, MD, USA) was used for electroporation and DNA propagation. Bacteria were grown in 2xYT medium [38]. For protein expression *E. coli* BL21(DE3) {F– *omp*T *hsd*SB(r_B–_, m_B–_) *gal dcm* (DE3)} were used (Life Technologies). The bacteria were cultivated in Terrific Broth (TB) medium [38]. Media were supplemented with chloramphenicol (40 μg/ml) and 0.2% maltose. Difco media components were obtained from Becton-Dickinson (Franklin Lakes, NJ). DEAE-cellulose and phosphocellulose P11 resin were purchased from Whatman (Springfield Mill, UK). Other chromatographic resins were from GE Healthcare (Uppsala, Sweden). The proofreading Marathon DNA Polymerase and DNA purification kits were from A&A Biotechnology (Gdynia, Poland). BspHI and SalI REases were from New England Biolabs (Ipswich, MA, USA). Protein standard, 100 bp DNA and 1 kb DNA markers were from Thermo Fisher Scientific/Fermentas (Vilnius, Lithuania). The cloning vector pRZ4737 (Cm^R^, P15A *ori*, *f*1 *ori*, *P*_*R*_ promoter) was from Bill Resnikoff [39]. T7 DNA was from Vivantis Technologies (Shah Alam, Malaysia). The DNA sequencing and PCR primer synthesis were performed at Vivantis Technologies and Genomed (Warsaw, Poland). All other reagents were purchased from Sigma-Aldrich (St. Louis, MO, USA).

### Sequencing, synthesis, amplification and cloning of wt-*taqIIRM* and syn-*taqIIRM* genes

#### Construction of the synthetic taqIIRM gene with low GC content

The *taqIIRM* gene nt sequence was obtained by a combination of sequencing of PCR products, prepared using a *T. aquaticus* genomic template and a proofreading DNA polymerase as well as direct genomic dideoxy and NGS sequencing approaches. Multiple runs of both strands were performed to ensure error-free determination of the high GC content in *T. aquaticus* DNA. Sequencing was performed through commercial services (Vivantis Technologies and Genomed). The codon-optimized synthetic gene was created using single strand (ss) 5′- overlapping complementary oligos with a length ranging from 40 to 60 nt. Both the top and bottom strand were covered with the phosphorylated ss oligos, subjected to ligation and PCR amplified using a proofreading DNA polymerase. The gene synthesis procedure was conducted by a commercial service at Vivantis Technologies.

#### Cloning of wt-taqIIRM and syn-taqIIRM genes

The approach to obtain overexpression of the TaqII bifunctional enzyme employed the modified vector pRZ4737, originally obtained from Bill Resnikoff [39] and further modified. The vector is a derivative of the pACYC184 plasmid [40], carrying a λ DNA section, containing the *P*_*R*_ promoter under the control of the CI repressor. The *cI* gene was located on the pRZ4737 backbone, allowing for host-independent expression in *E. coli*.

For gene cloning a ‘one-step DNA fragment assembly and circularization’ method was used [34]. The method recruits a thermostable DNA polymerase for the precise assembly of DNA overlapping fragments into circular constructs, under a low cycle number regime to minimize mutations. A linear vector backbone and the genes to be cloned were PCR amplified with proofreading Taq DNA polymerase blend using suitable oligos. DNA sequences of the primers used are in Table 2.

#### Linear vector backbone amplification

The PCR fragment, comprising the vector backbone was amplified from the modified pRZ4737 plasmid DNA [39,40], using FpRZ and RpRZ primers (Table 2). The PCR reaction was performed in 50 μl samples in a thermocycler (Biometra) and contained: 1× Marathon PCR Buffer, 0.1 mM of each dNTP, 0.5 μM of each primer, 1 ng of circular pRZ4737, and 0.25 units of proofreading DNA polymerase (Marathon DNA Polymerase). The PCR cycling profile for the linear vector backbone amplification was as follows: 94°C for 3 minutes (min), 80°C for 20 seconds (sec) (addition of DNA polymerase), 94°C for 30 sec, 67°C for 30 sec, and 72°C for 5 min (for 35 cycles); 72°C for 4 min.

#### *PCR amplification of the* wt*-taqIIRM and* syn*-taqIIRM* genes

The wt-*taqIIRM* gene was amplified from the *T. aquaticus* genomic DNA, using a PCR primer pair FTaq and RTaq, which introduced the following restriction sites: BspHI and SalI (after the TGA stop codon), respectively (Table 2).

The syn-*taqIIRM* gene was amplified from the original commercial fully synthetic gene DNA (Figure 1) using PCR primer pairs FsynTaq and RsynTaq, which introduced the restriction sites BspHI and SalI (after the TAG stop codon), respectively (Table 2). The 5’ ends of all the primers were complementary to the pRZ4737 DNA sequence (Table 2; DNA sequence fragments small letters).

The PCR reactions were performed in 50 μl samples in a thermocycler (Applied Biosystems) and contained: 1× Marathon PCR Buffer, 0.1 mM of each dNTP, 0.5 μM of each primer, either 0.5 ng syn-*taqIIRM* template DNA or 100 ng *T. aquaticus* genomic DNA, 3% DMSO and 0.2 units of DNA polymerase (Marathon DNA Polymerase). The PCR cycling profile for both the syn-*taqIIRM* and wt-*taqIIRM* gene amplification was as follows: 94°C for 3 min, 80°C for 20 sec (addition of DNA polymerase), 94°C for 30 sec, 67°C for 30 sec, and 72°C for 3.5 min (for 35 cycles); 72°C for 2 min.

#### Assembly of DNA fragments

DNA assembly and circularization was performed on non-purified PCR amplification products by high-fidelity PCR, in a single step. Each 50 μl sample contained 1× Marathon PCR Buffer, 0.1 mM of each dNTP, 100 ng of crude reaction product mix containing the linear vector backbone, 100 ng of crude reaction product mix including either the wt-*taqIIRM* or syn*-taqIIRM* gene, and 0.2 unit of Marathon DNA Polymerase. The molar ratios of insert to vector were 1.4 : 1.

The PCR cycling profile, optimized for DNA assembly, was as follows: 95°C for 3 min, 80°C for 20 sec (addition of DNA polymerase), 94°C for 30 sec, 58.5°C for 30 sec, and 72°C for 5 min (for 35 cycles); 72°C for 4 min. As the primers included complementary directional overhangs, the corresponding head and tail sequences of the vector and gene were annealed and assembled into plasmid pRZ-*taqIIRM* (Figure 3). After the assembly reaction, the methylated template pRZ4737 was subjected to DpnI digestion. The final DNA construct was phenol-chloroform extracted and ethanol precipitated. The resulting DNA was used to transform *E. coli* DH5α competent cells. After electroporation the bacteria were plated onto 2xYT medium supplemented with chloramphenicol (40 μg/ml) and 0.2% maltose at 28°C.

#### Selection of positive bacterial clones

Both SalI cleavage of plasmid DNA and direct PCR from a single bacterial colony were used for the screening of positives clones. After a preliminary analysis, plasmid DNA isolated from the selected bacterial clones was subjected to DNA sequencing. The promoter regions and the *taqIIRM* gene sequences (either wt or synthetic) of the recombinant plasmids were also confirmed.

### Expression of the recombinant wt and synthetic *taqIIRM* genes under *P*_*R*_ promoter in *E. coli*

The resulting positive clones were subjected to protein expression experiments. *E. coli* BL21(DE3) were electroporated either with pRZ-wt-*taqIIRM* or pRZ-syn-*taqIIRM* and mini-scale expression was performed by cultivation in 50 ml TB media supplemented with chloramphenicol and maltose at 28°C with vigorous aeration, followed by *P*_*R*_ promoter induction by a temperature shift to 42°C, when OD_600_ reached 0.9. The immediate temperature shift was obtained by the addition of 50 ml fresh TB medium, heated previously to 65°C. The cultivation temperature of 28°C was used to minimize residual TaqII REase activity, minimizing its toxicity for a bacterial host. It was anticipated that the temperature shift to 42°C promotes folding of the thermostable enzyme to its biologically active form. The culture growth was continued for 19 hours (h) at 42°C. Bacterial pellets from both the control, non-induced and induced cultures were subjected to SDS/PAGE electrophoresis. The gels were analysed for the appearance of the expected band size of ~120kDa [24]-125.7 kDa (this work) and for TaqII REase activity in crude lysates. The bacterial clones, efficiently expressing *taqIIRM* gene variants, were selected for a large-scale bacterial culture in a biofermentor.

### Purification of the recombinant TaqII enzyme

The recombinant TaqII purification procedure was common for both recombinant wt and synthetic gene-derived TaqII, and employed a simplified and modified protocol, which included some stages used for the native enzyme from *T. aquaticus*[35]. For large-scale protein purification, expression of both *taqIIRM* gene variants in *E. coli* BL21(DE3) [pRZ-wt-*taqIIRM* and pRZ-syn-*taqIIRM*] was initiated with bacteria inoculum washed out from a Petri dish into 1 L of rich TB media, supplemented with chloramphenicol at 28°C and 0.2% maltose. The culture was grown in a biofermentor Bioflo 115 (New Brunswick Scientific, Edison, NJ, USA) with vigorous aeration until OD_600_ reached 0.9, and then the λ promoter *P*_*R*_ was induced by a temperature shift to 42°C. The immediate temperature shift was obtained by the addition of fresh TB medium, heated previously to 65°C. After induction, the culture was supplemented with chloramphenicol and glucose to the final concentration of 0.2%. The induced bacteria were further cultivated at 42°C for 19 hours at 42°C. Having achieved an OD_600_ of 4.0, the culture was cooled down to 4°C and the cells were recovered by centrifugation. The yield was 48 g from 10 L of bacterial culture.

The purification scheme varied from the scheme described previously for native TaqII enzyme [35], and included the following stages (Figure 5):

1. *Lysis and heat treatment.* 48 g of bacterial cells was suspended in 4 volumes of buffer A [50 mM Tris–HCl (pH 7.5 at 25°C), 5 mM EDTA, 50 mM NaCl, 5% glycerol, 0.01% Triton-X-100, 0.01% Tween 20, 5 mM 2-mercaptoethanol (βMe), 0.5 mM PMSF, 1 mg/ml chicken egg lysozyme]. After 30 min incubation at 4°C, the lysate was centrifuged. The supernatant was supplemented with NaCl to a final 400 mM concentration, to reduce adsorption of remaining soluble proteins to the denatured fraction, and incubated for 30 min at 65°C. The denatured thermolabile *E. coli* proteins were removed by centrifugation.

2. *Polyethyleneimine (PEI) removal of nucleic acids.* PEI was gradually added to a clear lysate to 0.4%. Following 30 min stirring at 4°C, the nucleic acids/acidic proteins-PEI complexes were removed by centrifugation and the supernatant was subjected to ammonium sulphate (AmS) fractionation.

3. *AmS fractionation*. This stage was conducted in two phases. In the first step, 30% saturation was applied (at 4°C, 0.176 g/ml) and contaminating proteins were removed. In the second stage, 50% saturation was applied (additional 0.125 g/ml), the suspension was stirred overnight, centrifuged, dissolved in buffer B and dialysed against buffer B [20 mM K/PO_4_ (pH 8.0 at 25°C), 0.5 mM EDTA, 50 mM NaCl, 0.02% Triton X-100, 0.02% Tween 20, 5% glycerol, 10 mM βMe, 1 mM PMSF].

4. *Phosphocellulose P11 chromatography*. The separation was conducted in buffer B. As TaqII protein does not bind to the resin in the applied buffer conditions, it was used as a negative step. TaqII was eluted from the column in the flow-through and wash fraction, while contaminating proteins including residual non-specific nucleases were retained on the column. Both fractions were combined and dialysed against buffer C [20 mM Tris–HCl (pH 8.0 at 25°C), 0.5 mM EDTA, 30 mM NaCl, 0.01% Triton X-100, 0.01% Tween 20, 5% glycerol, 5 mM βMe, 0.1 mM PMSF].

5. *DEAE-Cellulose chromatography*. Anion exchange was conducted using buffer C with included increasing NaCl concentration steps in buffer C [mM]: 100, 150, 200, 250 and 500. TaqII was eluted at 150–200 mM NaCl. The DEAE-Cellulose chromatography was repeated twice. The second one was simplified and used for concentration of the TaqII protein. Column fractions containing the enzyme were dialysed against buffer C between repeated procedures and finally against buffer D [20 mM Tris–HCl (pH 8.3 at 25°C), EDTA, 25 mM KCl, 25 mM AmS, 0.05% Tween, 5 mM βMe, 3 mM MgCl_2_, 0.1 mM PMSF].

6. *Size exclusion chromatography on Sephadex G-100.* The procedure took advantage of the high molecular weight of TaqII REase as compared to other *E. coli* proteins. A Sephadex G-100 column was equilibrated in buffer D and concentrated TaqII preparation was subjected to molecular sieving. Purified preparation was dialysed against storage buffer S (20 mM Tris–HCl pH 8.3; 25 mM KCl; 25 mM AmS; 0.1 mM EDTA; 0.05% Triton X-100; 0.05% Tween 20; 0.5 mM DTT; 50% glycerol) and stored at −20°C.

### REase and MTase assays

For REase assays, the reactions were performed in 50 μl of ‘TaqII REase buffer’ (40 mM Tris–HCl pH 8.0 at 65°C; 1 mM DTT, 10 mM MgCl_2_, 10 mM AmS, bovine serum albumin (BSA) 100 μg/ml), supplemented with 100 μM SIN and DNA substrates. SIN was used as it is stable and highly stimulatory to TaqII REase. Addition of SIN simplified detection of the enzyme presence in the column fractions, which contained the enzyme inhibitory concentrations of salts and buffers, as well as boosted this inherently very ‘slow’ enzyme to allow more precise analysis.

One unit of the TaqII REase is defined for the purpose of this work as the amount of enzyme required to hydrolyse 1 μg of bacteriophage lambda DNA in 1 h at 65°C in 50 μl of TaqII REase buffer, enriched with 50 μM SIN, resulting in a stable partial DNA cleavage pattern.

The recombinant TaqII REase activity was investigated as described above at a temperature range from 40°C to 90°C. The pH of all the reaction buffers was determined at the appropriate reaction temperature.

The potential allosteric effectors were tested for stimulation of TaqII REase activity, using the TaqII REase assay described above. The incubation time was reduced to 30 min to obtain reaction conditions for partial DNA cleavage. The reactions were performed at 65°C in 50 μl of ‘TaqII REase buffer’ supplemented with 50 μM of SAM, SIN, SAH or ATP, respectively. A 390 bp PCR DNA fragment (containing two convergent TaqII sites 5′-GACCGA-3′ and ′CACCCA-3′) [23] was used as a DNA substrate. The reaction products were resolved on 15% poliacrylamide gel in TBE buffer and stained with Sybr Green I.

The *in vitro* modification activity of TaqII enzyme was tested by the DNA protection assay. The 390 bp PCR DNA fragment (containing single TaqII site 5′-GACCGA-3′) [23] was used as a substrate in 50 μl of TaqII MTase buffer (10 mM Tris–HCl pH 8.5 at 65°C; 1 mM DTT; 200 μM SAM) supplemented either with 10 mM CaCl_2_ or with 10 mM EDTA. After the addition of the TaqII protein, the reaction mixture was incubated for 16 h at 65°C. Proteinase K was added to the solution and the incubation was continued for additional 60 min at 55°C. Samples were purified to remove all traces of proteins and divalent cations from the methylation reaction mixture and the resulting DNA was challenged with an excess of TaqII (2:1 molar ratio of enzyme to recognition sites) for 1 h in 50 μl of TaqII REase buffer supplemented with 10 mM MgCl_2_ at 65°C. The reaction products were then resolved by agarose gel electrophoresis and TaqII MTase activity was assessed.

### Gel electrophoresis and protein concentration determination

#### DNA electrophoresis

1.5% agarose gels were prepared in TBE buffer [38]. The gels were visualized after staining with ethidium bromide using a 312 nm UV transilluminator. 15% polyacrylamide gels were prepared in 1x TBE buffer [38]. The gels were visualized after staining with SYBR Green I using a 312 nm UV transilluminator and photographed with a SYBR Green gel stain photographic filter.

#### Protein electrophoresis

SDS-PAGE electrophoresis of the proteins was in 10% polyacrylamide gels [38]. For the calibration curve, SDS-PAGE electrophoresis of various BSA concentrations was performed. Quantitative comparison of the resulting protein bands was made using UN-SCAN IT GEL for Windows 6.1 data software (v. 6.1, Gel Analysing and Graph Digitizing Software, Silk Scientific Corporation, Orem, Utah, USA). The calibration curve was used for the determination of the investigated TaqII protein variants concentration.

## Competing interests

The authors declare that they have no competing interest.

## Authors′ contributions

AZS designed most of the experiments and coordinated their execution, prepared all figures, co-drafted the manuscript and performed the experiments concerning cloning of the wt-*taqIIRM* gene. OZ performed the cloning and expression experiments of the syn-*taqIIRM* gene followed by syn-TaqII enzyme isolation and examined the enzymatic properties of both recombinant TaqII protein variants. KS isolated the recombinant wt-TaqII enzyme. JJF verified and corrected the wt *taqIIRM* contig sequence. PMS conceived the idea of *taqIIRM* synthetic gene cloning, designed its sequence, co-coordinated execution of the experiments, participated in the design and interpretation of experiments and co-drafted the manuscript. All authors read and approved the final manuscript.
